# Ingestion of Ubiquitous Environmental Metal‐Metalloid Neurotoxicants Lead, Cadmium, or Arsenic in Drinking Water Results in Brain Accumulation And Alters the Alzheimer’s Disease Exposome

**DOI:** 10.1002/alz.093261

**Published:** 2025-01-03

**Authors:** Lee E Goldstein, Kevin P Kotredes, Olga Minaeva, Juliet A Moncaster, Nicola Lupoli, Gareth R Howell

**Affiliations:** ^1^ Boston University Chobanian & Avedisian School of Medicine, Boston, MA USA; ^2^ Boston University Alzheimer’s Disease Research Center, Boston, MA USA; ^3^ The Jackson Laboratory, Bar Harbor, ME USA

## Abstract

**Background:**

Alzheimer’s disease (AD) and AD‐related dementias (ADRD) are modulated by gene‐environment (GxE) interactions across the lifespan. Variants of specific genes increase AD risk and synergize with lifetime exposure to environmental toxicants (“exposome”), including neurotoxic metals (lead, Pb; cadmium, Cd) and metalloid (As). These metal/metalloid toxicants readily enter the body (e.g., *via* contaminated drinking water), transit in blood, cross the blood‐brain barrier (BBB), and distribute in the brain. Pb, Cd, and As are potent neurotoxicants and suspected modifiers of AD pathobiology. The mechanisms underpinning AD‐related GxE interactions specific to Pb, Cd, As exposures are largely unknown and potentially modifiable. Hypothesis: Pb, Cd, As exposures alter expression of AD‐linked genes and potentiate AD pathogenesis in a toxicant‐specific, neurodevelopment‐sensitive, biomarker‐responsive, and age‐dependent manner.

**Method:**

We exposed mice expressing humanized AD gene variants (hAPOE, hMAPT, hAβ; NIA MODEL‐AD) to Pb, Cd, As in drinking water at human‐relevant toxicant levels (Pb: 200 ppm; Cd: 5, 50 ppm; As: 20 ppm) *vs* unspiked drinking water (control). Exposures were carried out for 30 days. Mice were sacrificed, blood collected by cardiac puncture, and cerebrovasculature cleared by transcardiac saline perfusion. Harvested brains were analyzed by ICP‐MS solution analysis, high‐resolution laser ablation metallomic imaging ICP‐MS mapping), transcriptomics, biochemistry, other assays.

**Result:**

MODEL‐AD mice exposed to Pb, Cd, As in drinking water increased levels of metal/metalloid toxicant in blood and brain and altered expression of human AD genes in a toxicant‐specific manner: decreased ↓*VGF* (Pb, As), increased ↑*APP* (Cd). Both ↓*VGF and* ↑*APP* are associated with increased AD risk. Pb distribution in brain showed striking regional variation. Significant associations (FDR<0.05; LOAD GO terms): Pb (600 genes): chaperone‐dependent protein refolding (FDR = 0.02), glutamate signaling (FDR = 0.009), cognition (FDR = 0.003), synaptic transmission (FDR = 0.01); As (201 genes): synaptic plasticity (FDR = 0.02) and organization (FDR = 0.04), unfolded protein response (FDR = 0.008); Cd (348 genes): protein localization (FDR = 0.03), protein metabolism (FDR = 0.05), sensory perception (FDR = 0.001). No group differences noted in water intake or daily weight.

**Conclusion:**

MODEL‐AD mice exposed to Pb, Cd, or As accumulate metal/metalloid toxicants in blood and brain and showed toxicant‐specific alterations in AD‐relevant gene expression and gene module profiles.

